# Mediterranean Diet Adherence, Nutrition Knowledge and Lifestyle Factors in Physically Active Adults and Athletes: The NUTRI_ACTIVE Study

**DOI:** 10.3390/nu18172810

**Published:** 2026-08-27

**Authors:** Simona Fiorini, Monica Guglielmetti, Lenycia de Cassya Lopes Neri, Ottavia Eleonora Ferraro, Mariangela Rondanelli, Anna Tagliabue, Cinzia Ferraris

**Affiliations:** 1 Laboratory of Food Education and Sport Nutrition, Department of Public Health, Experimental and Forensic Medicine, University of Pavia, 27100 Pavia, Italy; monica.guglielmetti@unipv.it (M.G.); lenyciadecassya.lopesneri@unipv.it (L.d.C.L.N.); cinzia.ferraris@unipv.it (C.F.); 2 Unit of Biostatistics and Clinical Epidemiology, Department of Public Health, Experimental and Forensic Medicine, University of Pavia, 27100 Pavia, Italy; ottavia.ferraro@unipv.it; 3 Human Nutrition and Eating Disorder Research Center, Department of Public Health, Experimental and Forensic Medicine, University of Pavia, 27100 Pavia, Italy; mariangela.rondanelli@unipv.it (M.R.); anna.tagliabue@unipv.it (A.T.)

**Keywords:** sport nutrition, mediterranean diet, athletes, energy availability, nutrition knowledge, lifestyle

## Abstract

**Background/Objectives**: Unhealthy diets and poor lifestyle choices can increase athlete’s risk of low energy availability (LEA) and its negative consequences. Conversely, optimal nutrition, such as the Mediterranean Diet (MedDiet), promotes health, recovery, and performance. This study evaluates MedDiet adherence among athletes and physically active individuals, exploring its relationship with nutrition knowledge (NK), dietary intake, eating behaviors, and sleep quality. **Methods**: This single-center observational study evaluated Italian healthy active adults/athletes (18–65 years) categorized into four performance Tiers. Validated questionnaires analyzed MedDiet adherence (MEDI-LITE), NK (GeSNK), eating behaviour (EAT-26 and TOS), sleep quality (PSQI), and physical activity (IPAQ). Dietary intake was estimated via a 7-day food diary. Body composition (skinfolds/BIVA), and resting energy expenditure (indirect calorimetry) were assessed. **Results**: Analysis included 169 participants (32.3 ± 11.9 years; 52.7% females; 56.8% Tier 1), practicing 2.0 ± 0.9 sports with 7.1 ± 3.7 h/week of training. Mean MEDI-LITE score was 10.6 ± 2.2 (moderate adherence) across all Tiers and was significantly, positively associated with NK (*p* = 0.002). Mean EA was 31.3 ± 8.6 kcal/kg FFM/day; sex-specific cut-offs revealed a substantial proportion of high-tier participants with low/suboptimal EA. Eating disorder risk and orthorexic tendencies were low, sleep quality was generally good; none significantly correlated with MEDI-LITE score. **Conclusions**: Higher NK was positively associated with MedDiet adherence. However, high-tier athletes showed low or suboptimal EA. Targeted nutrition education must integrate MedDiet principles with sport-specific fueling strategies, optimizing diet quality and energy adequacy.

## 1. Introduction

Optimal nutrition plays a central role in supporting both physical and mental well-being. Nutritional needs vary according to age, sex, type and intensity of physical activity, health status, and other individual factors. The scientific literature highlights the importance of a varied and well-balanced diet for athletes, whose nutritional requirements are shaped by personal characteristics, food preferences, training load, sport discipline, and competitive goal [1,2]. To sustain optimal physiological function, ensure appropriate macronutrient and micronutrient intake, support body composition goals, and promote adequate recovery, athletes’ diets must therefore be tailored to individual needs [1].

Appropriate consumption of carbohydrate (3–10 g/kg/day, according to the training load), protein (1.2–2.0 g/kg/day), and fat (20–35% of total energy intake) is equally essential and should be individualized and adjusted according to the athlete’s training program, competitive calendar, and metabolic characteristics [1]. Adequate hydration is another key component, with individualized fluid plans reflecting the characteristics of the training session or competitive event [3].

Athletes’ energy and nutrient requirements are influenced by numerous factors, including sex, sport typology, training intensity and duration, and environmental conditions such as heat, cold, or altitude [4]. At the same time, socioeconomic status, exposure to prior nutrition education, and nutrition knowledge (NK) may influence dietary choices and eating behaviours [5,6]. Together, these factors interact to shape food choices and ultimately influence dietary intake and nutritional adequacy. According to a recent systematic review, athletes with higher NK are more likely to translate that knowledge into healthier dietary behaviors [4], although not necessarily meeting the sport-related recommendations. In fact, despite some studies suggesting that athletes can meet caloric needs through a balanced diet [7], those engaged in high-volume or high-intensity training may struggle to achieve adequate energy intake [8]. This may lead to an increased risk of Low Energy Availability (LEA), a condition determining a wide range of physiological and psychological impairments, including muscle and weight loss, hormonal and menstrual dysfunction, reduced bone health, increased injury and illness risk, impaired recovery, and mental fatigue. These outcomes fall under the International Olympic Committee’s framework of Relative Energy Deficiency in Sport (REDs), which shows a considerable prevalence across sports, from 23 to 79.5% in females and 15–70% in males [9].

Parallel to performance-focused recommendations, several studies have demonstrated the benefits of the Mediterranean Diet (MedDiet) for overall health [10,11]. The MedDiet is a predominantly plant-based dietary pattern characterized by high consumption of whole grains, fruits, vegetables, legumes, nuts, herbs, spices, and olive oil, with moderate intake of fish, seafood, eggs, and dairy products, and limited consumption of red meat. Water is the main beverage, with moderate wine intake permitted with meals [12,13]. This dietary pattern has been associated with reduced risk of cardiovascular disease, certain cancers, and metabolic disorders. Although the underlying mechanisms remain partly unclear, several hypotheses point to lipid-lowering effects, improved oxidative and inflammatory status, modulation of hormones and growth factors, and favorable metabolic signaling [14]. Higher adherence to the MedDiet may therefore benefit athletes by supporting health, recovery, and long-term performance [15].

In the performance context, it is also important to evaluate other non-dietary factors potentially impacting recovery and overall health in physically active individuals and athletes. For example, adequate sleep duration and quality are essential to support cognitive function, metabolic regulation, immune function, and tissue repair, and to facilitate optimal training adaptations and performance [16,17]. Conversely, poor sleep quality and insufficient sleep have been associated with impaired performance, altered appetite regulation, increased injury risk, and negative effects on mental well-being [18]. Emerging evidence also suggests a bidirectional relationship between diet quality and sleep, with healthier dietary patterns, including higher adherence to the MedDiet [19,20]. However, data on the interaction between dietary patterns and sleep quality in athletic populations remain limited, highlighting the need for further investigation.

Given all these premises, mapping the nutritional status, dietary intake, eating behaviors, and sleep quality of physically active adults and athletes is essential to evaluate the adequacy of current lifestyle practices and to inform the development of targeted interventions. Specifically, the NUTRI_ACTIVE study primarily aims to assess adherence to the MedDiet among physically active adults and athletes and to explore its associations with nutrition knowledge, eating behaviours, anthropometric and body composition measures, and other lifestyle-related factors. In addition, the study describes dietary intake and energy availability (EA) across different levels of athletic participation, considering their potential relevance to nutritional adequacy and health.

## 2. Materials and Methods

This is a single-centre observational cross-sectional study. Participants were physically active adults and athletes contacted through local sport centres, amateur sports clubs, National Sport Federations, and the Laboratory of Nutrition Education and Sport Nutrition at the University of Pavia, Pavia, Italy, between July 2024 and October 2025. Individuals expressing interest in the project were enrolled after receiving information about the study and signing informed consent. The study was approved by the Territorial Ethics Committee Lombardia 6—Fondazione IRCCS Policlinico San Matteo (protocol no. 0006999/24, dated 5 February 2024). The Declaration of Helsinki principles were followed when conducting this study.

### 2.1. Participants

Eligible participants were physically active adults and athletes, of all genders, aged 18–65 years, engaged in regular physical activity, defined as at least 150 min of moderate-intensity or 75 min of vigorous-intensity aerobic activity per week, or an equivalent combination [21]. Individuals were excluded if affected by health conditions (i.e., diabetes, hypertension, dyslipidemia, metabolic syndrome, neurological diseases, …) or injuries that could influence nutritional status, metabolism or body composition.

All participants underwent a comprehensive nutrition assessment at the Laboratory of Nutrition Education and Sports Nutrition, Department of Public Health, Experimental and Forensic Medicine, University of Pavia, Pavia, Italy. Participants were screened to verify the inclusion and exclusion criteria and were classified according to the type of sport practiced: team sports, endurance, power, strength, precision, racquet sports, or combat/weight-making. Moreover, participants were categorised using the Classification Framework proposed by McKay et al. [22], which includes several levels, or Tiers, based on training volume and performance metrics. Tier 0 refers to sedentary individuals, while Tiers 1–5 describe different grades of physical activity, ranging from recreationally active individuals (Tier 1), to regularly trained/developmental athletes (Tier 2), highly trained national-level competitors (Tier 3), elite international athletes (Tier 4), and world-class performers (Tier 5). In the present study, only Tier 1 to Tier 5 were applicable, while the sedentary level (Tier 0) was not relevant given our inclusion criteria. Classification and participant selection were performed by trained professionals from the research team. Participants were also classified based on their nutritional knowledge background (e.g., having a Dietetics or Human Nutrition bachelor’s or master’s degree) into nutrition experts or non-nutrition experts.

During a 20–30 min interview, general information was collected, including age, gender, educational level, lifestyle and history, any previous or current nutrition interventions, and current use of dietary supplements.

### 2.2. Adherence to the Mediterranean Diet

Adherence to the MedDiet was evaluated using the MEDI-LITE score [23,24]. The questionnaire consists of nine multiple-choice items, each evaluating the frequency of consumption of different food groups: (1) fruit; (2) vegetables; (3) legumes; (4) cereals; (5) fish; (6) meat and meat products; (7) dairy products; (8) alcohol and (9) olive oil. For each food group, three response options are presented, differing according to the food group (e.g., <1 portion/day, 1–1.5 portion/day and >2 portion/day for fruit; <1 portion/week, 1–2 portion/week and >2 portion/week for legumes; “occasional use”, “frequent use” and “regular use” for olive oil). For food groups typical of the MedDiet (fruit, vegetables, cereals, legumes, and fish), 2 points were assigned to the highest consumption category, 1 point to the intermediate category, and 0 points to the lowest category. On the contrary, for food groups less typical of the MedDiet (meat and meat products, dairy products), 2 points were assigned to the lowest consumption category, 1 point to the intermediate category, and 0 points to the highest consumption category. The overall MEDI-LITE score, obtained by summing the points of all food groups, ranges from 0 (low adherence) to 18 (high adherence), with a MedDiet adherence optimal cut-off set at 8.5 [23,24].

### 2.3. Nutrition Knowledge

The General and Sport Nutrition Knowledge Questionnaire (GeSNK) is a validated instrument designed to assess NK across two domains: (1) general NK section comprises 29 items covering basic nutritional concepts, including nutrient composition, the relationship between diet and health, and principles of healthy eating; (2) sport NK section consists of 33 items addressing nutrition strategies before, during and after exercise, hydration and fluid replacement, sport supplements, and nutrition requirements related to physical activity [25].

Each correct answer is scored as 1 point, whereas incorrect or “I do not know” responses receive 0 points. Total scores range from 0 to 97, with higher values indicating greater NK. Since normative data for adults are not yet available, the classification of NK levels followed the scoring procedure proposed in the original validation study [25]. Specifically, we applied the percentile-based cut-offs derived from the adolescent and young adult sample as exploratory reference values: scores below the 33rd percentile were classified as low knowledge (General NK < 32, Sport NK < 14, total GeSNK < 46), those between the 33rd and 66th percentile as medium knowledge, and those above the 66th percentile as high knowledge (General NK > 40, Sport NK > 18, total GeSNK > 58).

### 2.4. Eating Behaviour

In addition to the adherence to MedDiet and NK evaluation, a set of validated questionnaires was administered to assess eating-related attitudes and orthorexic traits.

The Teruel Orthorexia Scale (TOS) is a validated Italian self-report instrument designed to distinguish between two dimensions of orthorexic eating behaviour [26]. The questionnaire consists of 17 items grouped into two subscales: Healthy Orthorexia (HeOr) (9 items), reflecting a non-pathological interest in healthy eating, and Orthorexia Nervosa (OrNe) (8 items), capturing an excessive preoccupation with dietary purity that may interfere with emotional, cognitive, and social functioning. Items are rated on a 4-point Likert scale (0 = completely disagree to 3 = completely agree). Subscale scores are derived by summing the relevant items, with higher values indicating stronger endorsement of each orthorexic dimension. No specific cut-off values have been reported by the authors. The TOS is widely used in both clinical and non-clinical populations to assess maladaptive forms of health-oriented eating.

The Eating Attitudes Test-26 (EAT-26) is one of the most widely used standardized instruments for screening symptoms and concerns related to eating disorders [27]. It comprises 26 items rated on a 6-point scale, generating a total score where higher values indicate greater eating-related psychopathology. A cut-off score ≥ 20 suggests the need for further clinical evaluation. However, low scores do not necessarily exclude disordered eating, as symptom denial may occur. The questionnaire has been extensively applied in both athletic and non-athletic populations.

### 2.5. Dietary Habits and Intake

Dietary habits were assessed through a structured individual interview conducted by trained investigators. The interview was designed to collect qualitative information on participants’ usual eating patterns and behaviors.

Specifically, data were collected on: (1) number of daily meals; (2) breakfast consumption; (3) meal skipping; (4) regularity of meal timing; (5) self-reported eating speed; (6) frequency of eating out; (7) current or previous adherence to specific dietary regimens; (8) use of dietary supplements.

Meal frequency was recorded as the average number of eating occasions per day. Breakfast consumption and meal skipping were assessed using dichotomous (yes/no) questions. Meal timing regularity was evaluated by asking participants whether meals were usually consumed at consistent times during the day. Eating speed was self-reported and categorized as slow, medium, or fast.

Eating out frequency was assessed as the number of meals consumed outside the home per week and categorized into predefined frequency classes. Participants were also asked whether they had followed a dietary regimen in the past or were currently following one at the time of the assessment. Dietary supplement use was recorded as a dichotomous variable (yes/no).

All information referred to habitual behaviors over the previous months and was collected prior to dietary intake assessment to avoid influencing participants’ responses.

Participants completed a 7-day weighted food diary to obtain a detailed record of the current dietary behaviour. The diary collected information about: (1) all foods and beverages consumed, including snacks and non-caloric items (e.g., water, coffee, tea); (2) quantities in grams/millilitres or standard household measures, specifying whether weights referred to raw or cooked foods; (3) brand and specific product type; (4) timing and place of consumption; (5) added condiments (e.g., oils, cheeses, sugar, sauces); and (6) ingredients and quantities for recipes (when applicable). Supplement use was also documented.

Dietary records were analysed to estimate daily energy intake as well as macro- and micronutrient intake using the Italian Food Composition Database for Epidemiological Studies as the reference food composition database [28].

Energy Availability (EA) was calculated as Energy Intake (EI) minus Exercise Energy Expenditure (EEE), normalized to Fat Free Mass (FFM). EA was expressed as kcal/kg FFM/day. Participants were categorized into EA classes based on current reference thresholds. For female participants, values ≥ 45 kcal/kg FFM/day were considered indicative of adequate EA, values between 30 and 44.9 kcal/kg FFM/day were classified as suboptimal, and values < 30 kcal/kg FFM/day were considered LEA. For male participants, given the lack of established sex-specific EA thresholds, the categories were operationalized based on the range of approximately 9–25 kcal/kg FFM/day. Values < 9 kcal/kg FFM/day were classified as LEA, values between 9 and 25 kcal/kg FFM/day as sub-optimal EA, and values > 25 kcal/kg FFM/day as adequate EA [9].

Carbohydrate availability (CA) was evaluated by comparing individual daily carbohydrate intake, expressed as g/kg body weight, with sport-specific recommendations based on weekly training volume. Reference carbohydrate requirements were defined according to guidelines for sports nutrition [1], as follows: 3–5 g/kg/day for individuals training 3–5 h/week; 5–7 g/kg/day for 5–7 h/week; 6–10 g/kg/day for 7–21 h/week; 8–12 g/kg/day for individuals training ≥4–5 h/day. Participants whose carbohydrate intake fell below the recommended range for their respective training volume were classified as having low CA (LCA).

### 2.6. Sleep Quality

The Pittsburgh Sleep Quality Index (PSQI) is a validated self-report measure evaluating sleep quality over the preceding month [29]. The questionnaire includes seven components: subjective sleep quality, sleep latency, sleep duration, sleep efficiency, sleep disturbance, sleep medication use, and diurnal dysfunction. These components yield a global score ranging from 0 to 21, with higher scores indicating poorer sleep quality. Specifically, sleep quality was categorised as poor if the PSQI score was  ≥5 and good if it was  <5.

### 2.7. Physical Activity Level

The International Physical Activity Questionnaires (IPAQ)—long form questionnaire has also been administered to the participants for physical activity duration and frequency assessment in the last seven days. This questionnaire analyses five domains: (1) job-related activity; (2) transportation; (3) housework, house maintenance, caring for family; (4) recreation, sport and leisure time; and (5) time spent sitting [30]. Due to the different participants’ distribution across the Tiers, it was not possible to check any potential differences in the studied characteristics among the Tiers’ participants.

### 2.8. Anthropometric and Body Composition Evaluation

Anthropometric assessments included body weight, stature, circumferences, skinfold thicknesses, and bioelectrical impedance vector analysis (BIVA).

Body weight and height were measured using a mechanical scale equipped with an integrated stadiometer. Participants were assessed barefoot and wearing light clothing (e.g., underwear). Height was recorded with the participant standing erect, heels, buttocks, and scapulae aligned with the vertical stadiometer bar, and head positioned in the Frankfurt plane to minimize parallax error.

Two anatomical circumferences were measured using a non-elastic tape: (1) waist circumference at the umbilical level; (2) hip circumference at the level of the greater trochanters. From these measurements, the following anthropometric indices were derived: Body Mass Index (BMI) (kg/m^2^); Waist-to-Hip Ratio (WHR), an indicator of body fat distribution and cardiometabolic risk (values > 0.8 in women and >1.0 in men); Waist-to-Height Ratio (WHtR), considered a sensitive predictor of metabolic risk (values > 0.5).

Skinfold thicknesses were measured using a calibrated skinfold caliper. Eight sites (subscapular, suprailiac, biceps, triceps, axillary, pectoral, abdominal, thigh) were assessed in triplicate but the mean 7-skinfolds value was used for analysis [31,32]. Body density was estimated using predictive equations based on skinfold thickness measurements [31,32]. Body density (BD) was subsequently converted to Fat Mass % (FM) using Siri’s equation (FM% = (495/BD) − 450).

Whole-body composition was evaluated using a BIA 101 BIVA Pro device (Akern), a vector impedance analyzer based on a 50 kHz sinusoidal current. The tetrapolar electrode configuration (hand-foot) was applied on the right side of the body using Ag/AgCl electrodes (Biatrodes, Akern). Measurements were taken in the supine position, with limbs slightly abducted and the body isolated from conductive surfaces. The device records resistance (R) and reactance (Xc), which reflect total body water, fluid distribution, and cellular membrane integrity. Total Body BIVA estimated FFM, FM, and phase angle (PhA°), a marker of cell integrity and metabolic health.

Resting Energy Expenditure (REE) was measured using indirect calorimetry, considered the gold standard for assessing basal metabolic rate under fasting, thermoneutral, and fully rested conditions. The method estimates energy expenditure through oxygen consumption (VO_2_) and carbon dioxide production (VCO_2_), applying the Weir equation, which does not require urinary nitrogen collection. The respiratory quotient (RQ = VCO_2_/VO_2_) was also derived to provide qualitative information on substrate oxidation [33]. Measurements were performed using a canopy system (Q-NRG^®^, COSMED, Rome, Italy). Participants fasted for at least 8 h and were instructed to refrain from any physical activity for the 24 h prior to the measurement. Measurements were conducted in the morning (around 8 or 9 a.m.), with participants resting in a supine position in a quiet, thermoneutral environment to standardize the assessment. Each assessment lasted approximately 20 min, during which expired gases were continuously collected through a transparent canopy.

### 2.9. Statistical Analysis

Descriptive statistics were used to summarize the baseline characteristics of the sample. Continuous variables are presented as mean and standard deviation, while categorical variables are presented as frequencies and percentages. Given the exploratory nature of the study, separate univariable linear regression models were performed to examine the association between each nutritional, anthropometric, and behavioral characteristic and the MEDI-LITE score. No adjustment for potential confounders or covariates was performed.

Finally, due to the small number of participants in some Athlete Tier categories, comparisons across Athlete Tiers were limited to descriptive analyses, and no inferential tests were performed. Two-sided *p*-values were reported for the univariable linear regression models, with *p* < 0.05 considered statistically significant. Statistical analyses were performed using Stata^®^ v.19.

## 3. Results

### 3.1. Participants’ Characteristics

A total of 169 Italian physically active individuals and athletes were included in the analysis. Participants had a mean age of 32.3 ± 11.9 years. The sample was composed of 52.7% females (*n* = 89) and 47.3% males (*n* = 80). No cases of gender dysphoria were reported. The majority of participants were classified as Tier 1 (*n* = 96, 56.8%), followed by Tier 2 (*n* = 42, 24.9%), Tier 3 (*n* = 26, 15.4%), and Tier 4 (*n* = 5, 3.0%). Given the sample size and the distribution of participants across multiple Tier categories, with some groups including a limited number of individuals, formal statistical comparisons between Tiers were not performed. The small cell counts would have resulted in limited statistical power and potentially misleading inferences.

Most participants resided in Northern Italy (93.5%), with a smaller proportion from Central (3.0%) and Southern regions (3.6%). Regarding marital status, 74.6% of the sample were single/unmarried while 25.4% were married. The majority of participants did not have children (84.0%). Educational level was generally high, with 67.4% holding a university degree (bachelor, master or doctorate) and 31.4% a high school diploma. 23.1% of the sample reported a nutritional knowledge background. Based on lifestyle interviews, most participants were non-smokers (85.2%), while 14.2% reported current smoking. Alcohol consumption was reported by 67.5% of the sample, with a low average intake (0.1 ± 0.2 alcohol units/day).

When stratified by Tier, sex distribution differed across groups, with a higher proportion of males observed in Tier 2 (54.8%) and Tier 3 (65.4%), while Tier 1 and Tier 4 showed a higher prevalence of females. Mean age was comparable across Tier 1, 2, and 3 (31.2 ± 10.4, 35.2 ± 13.8, and 33.5 ± 13.3 years, respectively), whereas Tier 4 participants were younger (21.7 ± 3.4 years).

Demographic characteristics of the study sample, overall and stratified by Tier, are detailed in Table 1.

### 3.2. Mediterranean Diet Adherence, Nutrition Knowledge, Eating Behaviour and Sleep Quality

The main questionnaire-based outcomes, including MedDiet adherence, nutrition knowledge, eating behaviours, and sleep quality, were assessed in the overall study population and across Athlete Tiers (Table 2).

Adherence to the MedDiet, assessed using the MEDI-LITE score, averaged 10.6 ± 2.2 in the total sample. Similar values were observed across Tiers, with scores ranging from 9.9 ± 2.0 in Tier 3 to 10.8 ± 2.3 in Tier 2 and 10.8 ± 2.2 in Tier 4 (Table 2).

In the overall sample, the mean GeSNK score was 72.8 ± 14.9, with Tier-specific mean values ranging from 70.2 ± 19.1 in Tier 2 to 78.1 ± 8.4 in Tier 3. General NK showed a mean score of 49.4 ± 7.8 in the total sample, with mean values of 51.3 ± 6.9 in Tier 3 and 41.8 ± 6.3 in Tier 4. Sport-specific NK averaged 24.7 ± 4.8 overall, with mean values ranging from 24.1 ± 5.2 in Tier 1 to 26.8 ± 3.0 in Tier 3, and a mean value of 23.4 ± 5.5 in Tier 4.

Eating behaviors were evaluated using the TOS and EAT-26 questionnaires. In the total sample, mean HeOr score was 14.4 ± 5.0, with a mean value of 16.4 ± 5.9 in Tier 3. OrNe scores were generally low across the sample, with a mean value of 4.8 ± 3.6, showing minimal variation across Tiers.

The EAT-26 score averaged 4.4 ± 5.2 in the total sample, with mean values of 5.1 ± 4.9 in Tier 3 and 2.6 ± 1.7 in Tier 4. Only 4 participants were identified as having an EAT-26 score above the cut-off for increased risk of eating disorders (2.4%).

The mean PSQI score in the overall sample was 3.8 ± 2.4. Tier-specific mean scores, observed across Tier classifications, were 3.7 ± 2.3 in Tier 1, 4.0 ± 3.2 in Tier 2, 4.1 ± 1.7 in Tier 3, and 3.2 ± 1.1 in Tier 4.

### 3.3. Dietary Habits

Dietary habits were assessed through a structured individual interview and are summarized for the whole sample and by Tier (Table 3). Overall, participants reported consuming an average of 4.3 ± 0.9 meals per day, with Tier-specific values reported in Table 3.

The majority of the sample reported regular breakfast consumption (94.7%, *n* = 160), although 65.7% reported consuming meals at irregular times. 16.6% of the sample reported skipping meals. Eating speed was predominantly reported as medium (36.7%) or fast (47.3%), whereas only 16.0% reported eating slowly. Eating out frequency varied considerably, with 26.3% of participants reporting eating out twice per week. Higher frequencies (≥3 times/week) were reported by approximately 13.8% of the sample.

Regarding dietary practices, 52.7% of participants reported having followed a diet in the past, while 18.3% were currently following a dietary regimen.

The use of dietary supplements was reported by 55.6% of participants, with Tier-specific percentages reported in Table 3 and a value of 100.0% in Tier 4.

### 3.4. Dietary Intake

Dietary information was available only for 145 individuals. Dietary intake was assessed through the 7-day food diary and is detailed in Table 4. Most participants followed an omnivorous diet (95.0%), with only a small proportion reporting vegetarian (3.5%) or vegan (1.4%) dietary patterns.

Mean daily energy intake for the whole sample was 2131.7 ± 508.2 kcal/day, with tier-specific mean values reported in Table 4. EA, expressed as kcal/kg FFM/day, averaged 31.3 ± 8.6 kcal/kg FFM/day in the total sample. Mean EA values were 26.1 ± 11.0 kcal/kg FFM/day in Tier 3 and 23.4 ± 17.6 kcal/kg FFM/day in Tier 4.

Mean protein intake was 96.3 ± 27.9 g/day, corresponding to 1.4 ± 0.4 g/kg body weight and 18.1 ± 3.9% of total energy intake. Tier-specific mean protein intakes are reported in Table 4, with the highest numerical value observed in Tier 4.

Mean carbohydrate intake was 284.2 ± 79.4 g/day, accounting for 50.2 ± 5.2% of total energy intake and 4.1 ± 1.2 g/kg body weight. Absolute and relative carbohydrate intakes showed numerically different values across Tier categories, as reported in Table 4. Mean fat intake was 72.5 ± 19.7 g/day, corresponding to 30.7 ± 4.4% of total energy intake. Saturated fat intake accounted for 9.8 ± 2.3% of energy intake, while polyunsaturated and monounsaturated fatty acids contributed 4.1 ± 0.9% and 14.7 ± 11.5%, respectively. Mean fiber intake was 27.7 ± 7.6 g/day, while cholesterol intake averaged 223.2 ± 77.5 mg/day. Mean daily water intake from beverages was 2.2 ± 0.7 L, with higher values observed in higher Tiers. Mean daily intakes of micronutrients are specified in Table 4.

### 3.5. Physical Activity

Physical activity and sport-related characteristics of the sample, overall and stratified by Tier, are reported in Table 5. Participants practiced a mean of 2.0 ± 0.9 sports, with 31.4% reporting participation in a single sport, 44.4% in two sports, and 24.2% in three or more sports. The proportion of participants engaged in three or more sports was numerically higher in Tier 3 and Tier 4 than in Tier 1 and Tier 2.

Regarding sport type, a wide range of disciplines was represented. In the overall sample, strength (37.9%), endurance (29.0%), and team-based (11.8%) sports were the most frequently reported primary sport categories, followed by power (3.6%), racquet (3.0%), combat (2.4%), precision (5.9%), and other sports (6.5%). The descriptive distribution of sport types varied across Tiers. Tier 1 included a greater proportion of participants engaged in recreational and fitness-oriented activities, whereas endurance- and power-based sports were more frequently represented in Tier 2 and Tier 3.

Mean weekly training volume in the total sample was 7.1 ± 3.7 h/week, with Tier-specific values of 5.3 ± 2.4 h/week in Tier 1, 8.5 ± 3.1 h/week in Tier 2, 9.6 ± 3.8 h/week in Tier 3, and 15.4 ± 6.5 h/week in Tier 4. Average daily training time was 0.8 ± 0.3 h/day in Tier 1, 1.2 ± 0.4 h/day in Tier 2, 1.4 ± 0.5 h/day in Tier 3, and 2.2 ± 0.9 h/day in Tier 4. In the overall sample, participants reported 5.2 ± 2.3 training sessions per week, with Tier-specific mean values ranging from 4.4 ± 1.9 sessions/week in Tier 1 to 9.5 ± 0.6 sessions/week in Tier 4.

Previous sport-related injuries were reported by 40.5% of the total sample. The proportions reporting previous injuries were 31.6% in Tier 1, 42.9% in Tier 2, 57.7% in Tier 3, and 100% in Tier 4.

The overall IPAQ score was 8253.4 ± 5470.0 MET·min/week, indicating high levels of physical activity in our sample. Tier-specific mean IPAQ scores were 6767.2 ± 3047.2 MET·min/week in Tier 1, 9677.0 ± 7878.6 in Tier 2, 10,793.0 ± 6507.4 in Tier 3, and 12,876.2 ± 5102.6 MET·min/week in Tier 4 (Table 5).

### 3.6. Anthropometry and Body Composition

Anthropometric characteristics and body composition parameters of the subjects enrolled, overall and stratified by Tier, are reported in Table 6.

In the overall sample, mean body mass was 70.7 ± 12.4 kg, mean height was 1.7 ± 0.1 m, and mean BMI was 23.7 ± 2.9 kg/m^2^, with Tier-specific values reported in Table 6. Body composition assessment revealed a mean body fat percentage of 18.6 ± 6.6% estimated from skinfold measurements and 22.4 ± 7.3% assessed by bioelectrical impedance analysis. When considering the average value derived from both methods, mean FM percentage was 20.5 ± 6.6%, with Tier-specific mean values ranging from 21.8 ± 6.8% in Tier 1 to 18.3 ± 5.2% in Tier 4. Mean FM expressed in kilograms and FFM values across Tier categories are reported in Table 6. Women had a mean FM percentage of 24.3 ± 6.0%, compared with 16.3 ± 4.2% in men, while sex-specific FFM values are reported in Table 6.

Moreover, 14 participants (8.3%) were classified as having elevated FM% (>32% for female, >25% for male), whereas only one female participant fell below the essential fat category for females (10–13%).

Indicators of central adiposity showed that most participants were not at cardiometabolic risk, with 79.9%, 82.2%, and 74.0% classified as not at risk according to waist circumference, WHR, and WHtR, respectively. Tier-specific proportions of participants classified as at risk are reported in Table 6. Bioelectrical parameters indicated a mean phase angle of 6.4 ± 0.7° in the overall sample, with Tier-specific mean values reported in Table 6.

Resting metabolic rate measured by indirect assessment averaged 1621.2 ± 315.7 kcal/day, with Tier-specific mean values reported in Table 6.

### 3.7. Mediterranean Diet Adherence and Associated Variables

Associations between the MEDI-LITE score and nutritional, anthropometric, behavioral, and sport-related variables are presented in Table 7.

The MEDI-LITE score was significantly and positively associated with NK. Specifically, each one-unit increase in the GeSNK score was associated with a 0.04-point increase in the MEDI-LITE score (β = 0.04, 95% CI 0.02 to 0.07, *p* = 0.002). Similarly, significant positive associations were observed for General NK (β = 0.08, 95% CI 0.04 to 0.12, *p* < 0.001) and Sport NK (β = 0.09, 95% CI 0.02 to 0.16, *p* = 0.009).

No significant associations were observed between the MEDI-LITE score and eating disorder risk, orthorexic tendencies, physical activity levels, or sleep quality.

With regard to body composition, the MEDI-LITE score was positively related to FM% (β = 0.07, 95% CI 0.02 to 0.12, *p* = 0.011), while a significant inverse association was observed with fat-free mass (FFM, kg) (β = −0.06, 95% CI −0.09 to −0.03, *p* < 0.001). No significant associations were found between MEDI-LITE and BMI or WHtR.

EA showed a small but statistically significant positive association with the MEDI-LITE score (β = 0.04, 95% CI 0.00 to 0.08, *p* = 0.045). When EA categories were considered, adequate EA was not significantly associated with the MEDI-LITE score compared with LEA (β = 0.05, 95% CI −0.78 to 0.88, *p* = 0.904). In contrast, suboptimal EA was significantly associated with a lower MEDI-LITE score compared with LEA (β = −1.80, 95% CI −3.49 to −0.10, *p* = 0.038).

Interestingly, participants currently following a nutrition plan showed a lower estimated MEDI-LITE score than those not following a nutrition plan, although the association was not statistically significant (β = −0.92, 95% CI −1.86 to 0.02, *p* = 0.056).

No other dietary habit variables or previous dieting behaviors were significantly associated with the MEDI-LITE score.

## 4. Discussion

The NUTRI_ACTIVE study provides a comprehensive overview of MedDiet adherence, nutritional status, dietary intake, eating behaviors, and NK in a sample of physically active adults and athletes of different disciplines and training levels. The sample was characterized by a relatively young age, a high educational level, and generally health-oriented lifestyle habits, including low smoking prevalence and low alcohol consumption. These characteristics likely influenced both the overall dietary patterns observed and the low prevalence of dysfunctional eating behaviors in the sample, and should be considered when interpreting the findings.

Overall adherence to the MedDiet was moderate (10.6 ± 2.2) and showed limited variability across participants from different Tiers. Despite marked differences in training volume, physical activity levels, and sport typology, MedDiet showed similar values across recreationally active individuals and higher-level athletes. This finding suggests that increasing athletic involvement does not necessarily translate into greater adherence to a high-quality dietary pattern. Similar findings have been reported in previous studies conducted in athletic populations, including those living in Mediterranean countries, where adherence to the MedDiet generally appears to be moderate rather than optimal [34,35,36,37]. For instance, Costantino and colleagues reported that less than half of physically active individuals adhered to the core principles of the MedDiet [38], while the systematic review by Calella et al. highlighted that most studies in sport settings describe a medium level of adherence, despite more favorable patterns compared with inactive controls [39]. Athletes frequently prioritize performance-oriented nutritional strategies, such as macronutrient distribution and timing, which may not fully align with traditional dietary patterns [40].

One of the most consistent findings of this study is the positive association between MedDiet adherence and NK, including general, sport-specific, and overall knowledge scores. Although a small increase in the MEDI-LITE score is reported for each 1-point increase in NK, these results support previous evidence indicating that higher NK is associated with better diet quality and healthier food choices [4,6]. Interestingly, NK scores were relatively high across all Tiers, with only small differences between groups. This may reflect the high educational level of the sample and the widespread availability of nutrition information among physically active individuals. However, the modest strength of these associations suggests that NK alone may not be sufficient to ensure optimal dietary patterns, particularly in athletic populations.

In the present study, the interpretation of EA is better supported by prevalence data rather than by mean values. Given the well-documented sex-specific thresholds for EA, average values calculated in mixed-sex samples may obscure clinically relevant conditions, particularly in populations characterized by heterogeneous training loads and body composition profiles. Further analyses stratified by sex may help to clarify potential differences in the distribution of EA categories and their associations with dietary patterns, an aspect that warrants specific investigation in future studies.

When EA was categorized using sex-specific cut-offs, a substantial proportion of participants were classified as having low or suboptimal EA. This finding is consistent with previous literature reporting a high prevalence of inadequate EA among physically active individuals and athletes, even in the absence of overt signs of disordered eating/eating disorders or low body weight [9,41,42]. This suggests that EA may be influenced not only by food choices but also by dietary timing, portion size, and performance-driven nutritional strategies, which may partially explain the complex relationship observed between EA and adherence to healthy dietary patterns in athletic populations.

In line with this interpretation, the analysis based on EA categories revealed that participants classified as having suboptimal EA exhibited significantly lower MEDI-LITE scores compared with those with LEA, whereas no significant differences were observed between participants with adequate and LEA. This counterintuitive result may reflect compensatory dietary behaviors, whereby individuals with LEA maintain relatively higher diet quality but insufficient overall energy intake. Alternatively, athletes with suboptimal energy availability may adopt more restrictive or performance-focused dietary strategies that reduce adherence to the MedDiet. These findings highlight the complexity of the relationship between diet quality and energy intake in athletic populations and underscore the importance of evaluating both dietary patterns and quantitative intake when assessing nutritional adequacy.

Overall scores for eating disorder risk and orthorexic tendencies were low in the present sample, with slightly higher values observed in participants from intermediate Tiers. These results indicate a generally low prevalence of disordered eating behaviors in this cohort of physically active adults and athletes. This observation aligns with the heterogeneity reported in the literature, where prevalence estimates of eating disorders in athletic populations vary widely according to sex, sport discipline, and competitive level. Available data suggest prevalence ranges from 0% to 19% in male athletes and from 6% to 45% in female athletes, with higher values observed in aesthetic and endurance sports compared with the general population [43]. For instance, a recent cross-sectional study on adult athletes in Italy and Lebanon reported a higher risk of eating disorder, assessed using the EAT-26 questionnaire, equal to 6.1% in Italian athletes (7.8% females, 4.9% males) and 21.3% in Lebanese athletes (27.3% females, 17.0% males) [44].

In the present study, no significant associations were found between MEDI-LITE score and either eating disorder risk or orthorexic tendencies. These findings suggest that adherence to this dietary pattern was not accompanied by any modification in the risk of maladaptive eating behaviors in physically active individuals and athletes. This is consistent with results reported by Muros and colleagues in a large sample of Spanish cyclists and triathletes, where MedDiet adherence did not predict the likelihood of disordered eating, despite other demographic and behavioral factors being significant predictors [45]. In contrast, Martinovic et al. observed a significant negative correlation between MedDiet adherence and ORTO-15 scores in both professional (r = −0.365, *p* < 0.001) and recreational (r = 0.309, *p* < 0.001) athletes, suggesting that higher adherence to a Mediterranean-style dietary pattern was associated with greater orthorexic tendencies [34]. Conversely, studies conducted in non-athletic populations have suggested a potential protective role of the MedDiet against the incidence of eating disorders, including anorexia and bulimia nervosa [46]. Differences in study populations, assessment tools, and outcome measures likely contribute to these inconsistent findings. In the present study, eating disorder risk and orthorexic tendencies were assessed using self-reported screening questionnaires, which capture risk and behavioral tendencies rather than clinically diagnosed disorders. Moreover, the overall low scores observed across the sample limited the variability required to detect significant associations.

The relatively low scores observed may reflect the recreational or sub-elite nature of most participants, as well as the known limitations of self-reported instruments, which are susceptible to underreporting and social desirability bias [43]. Longitudinal and sport-specific studies are therefore warranted to clarify whether adherence to specific dietary patterns influences the development of eating disorders or orthorexia over time, particularly in high-risk subgroups such as female athletes involved in aesthetic or endurance sports [47].

Sleep quality, assessed using the PSQI, was generally good across all Tiers, with no significant differences observed between groups. The lack of association between dietary pattern quality and sleep quality contrasts with findings from general population studies, where Mediterranean-style diets have been linked to better sleep outcomes [20,48]. In athletic populations, however, sleep quality is influenced by multiple sport-specific factors, such as training schedules, competition stress, and travel demands, which may attenuate potential dietary effects.

Several limitations of the present study should be acknowledged. First, the cross-sectional design does not allow causal inferences to be drawn regarding the relationships between MedDiet adherence, EA, NK, eating behaviors, and sleep quality. Although significant associations were identified, longitudinal or intervention studies are required to clarify the directionality of these relationships. Furthermore, the analyses examining associations between MedDiet adherence and multiple nutritional, anthropometric, and behavioral variables were exploratory, and no formal correction for multiple comparisons was applied. Therefore, these findings should be interpreted in light of the exploratory nature of this study and should be further evaluated in future confirmatory studies. Second, dietary intake and eating habits were assessed using self-reported methods, including a 7-day food diary and structured interviews. While the 7-day diary is commonly used in sports nutrition research and allows detailed assessment of habitual intake, self-reporting is inherently subject to recall bias, misreporting, and social desirability bias, particularly in physically active individuals and athletes who may be more aware of nutritional recommendations [49]. In addition, under-reporting of energy intake may have influenced the estimation of EA, potentially leading to misclassification in EA categories. Third, the assessment of EA itself represents a methodological challenge. Despite the use of indirect calorimetry to assess resting energy expenditure, EA was estimated using indirect measures of energy intake and exercise energy expenditure, which may introduce error. Moreover, although sex-specific cut-offs were applied, EA remains a dynamic construct that can fluctuate substantially across training cycles, competition periods, and recovery phases. A single assessment period may therefore not fully capture chronic exposure to low or suboptimal EA. Fourth, our sample was predominantly composed of young and highly educated individuals residing in Northern Italy, which may limit the generalizability of the findings to other geographic regions, age groups, or populations with different socioeconomic backgrounds. Additionally, although participants were classified across different Tiers, the relatively small number of individuals in the highest Tier reduced statistical power for comparisons involving elite athletes and may have limited the detection of Tier-specific associations. Given the recruitment strategy and sample characteristics, the findings of this study are primarily applicable to physically active adults and athletes engaged in structured sport settings, and may be less generalizable to different sociocultural contexts. Fifth, sleep quality was assessed using the Pittsburgh Sleep Quality Index, a validated and widely used questionnaire. However, subjective measures of sleep may not fully reflect objective sleep parameters such as sleep duration, efficiency, or fragmentation. The generally good sleep quality observed in the sample may have limited the ability to detect meaningful associations with dietary patterns.

Despite its cross-sectional design, the present study offers several strengths that enhance the interpretation and relevance of the findings. A key strength is the comprehensive assessment of nutritional and lifestyle factors within a single cohort of physically active adults and athletes. By evaluating MedDiet adherence, dietary habits and intake, energy and carbohydrate availability, NK, eating behaviors, sleep quality, and sport participation characteristics, the study provides an integrated picture that reflects the complexity of real-world sport nutrition contexts. Another important strength is the inclusion of participants across different competitive Tiers. This approach allows dietary behaviors and nutritional risk factors to be interpreted along a continuum of sport involvement. Finally, the assessment of EA, combined with the evaluation of carbohydrate availability based on sport-specific recommendations, strengthens the clinical and practical relevance of the findings. Collectively, these elements support the robustness of the study and contribute novel insights into the relationship between dietary patterns, nutritional adequacy, and health-related behaviors in physically active populations.

## 5. Conclusions

The findings of the NUTRI_ACTIVE study highlight the positive association between nutrition knowledge and MedDiet adherence in physically active individuals and athletes, underscoring the importance of addressing both dietary quality and energy adequacy in physically active individuals and athletes. Nutrition interventions should aim to integrate MedDiet principles with sport-specific fueling strategies, rather than treating these approaches as mutually exclusive.

Future studies should adopt longitudinal or interventional designs to evaluate whether targeted nutrition intervention can improve dietary pattern quality while maintaining adequate EA. Further research exploring sport-specific and sex-specific differences may also contribute to more tailored and effective nutritional recommendations.

## Figures and Tables

**Table 1:** Demographic characteristics of the study population, overall and stratified by Tier.

Participants Characteristics					
	Total (*n* = 169)	Tier 1 (*n* = 96)	Tier 2 (*n* = 42)	Tier 3 (*n* = 26)	Tier 4 (*n* = 5)
Age (years) ^a^	32.3 ± 11.9	31.2 ± 10.4	35.2 ± 13.8	33.5 ± 13.3	21.7 ± 3.4
Female, *n* (%)	89 (52.7)	58 (60.4)	19 (45.2)	9 (34.6)	3 (60.0)
*Area of residence*					
North, *n* (%)	158 (93.5)	87 (90.6)	41 (97.6)	26 (100.0)	4 (80.0)
Center, *n* (%)	5 (3.0)	4 (4.2)	0 (0.0)	0 (0.0)	1 (20.0)
South, *n* (%)	6 (3.6)	5 (5.2)	1 (2.4)	0 (0.0)	0 (0.0)
*Marital status*					
Unmarried/single, *n* (%)	126 (74.6)	76 (79.2)	29 (69.1)	16 (61.5)	5 (100.0)
Married/partner, *n* (%)	43 (25.4)	20 (20.8)	13 (31.0)	10 (38.5)	0 (0.0)
Having children, *n* (%)	26 (16.0)	12 (13.0)	7 (17.5)	7 (26.9)	0 (0.0)
*Education*					
High school graduation or lower, *n* (%)	55 (32.6)	23 (24.0)	12 (28.6)	16 (61.5)	4 (80.0)
University degree, *n* (%)	106 (62.7)	69 (71.9)	26 (61.9)	10 (38.5)	1 (20.0)
Doctorate, *n* (%)	8 (4.7)	4 (4.2)	4 (9.5)	0 (0.0)	0 (0.0)
Nutrition experts, *n* (%)	39 (23.1)	25 (26.0)	8 (19.0)	5 (19.2)	1 (20.0)
Smoker, *n* (%)	24 (14.2)	12 (12.5)	8 (19.0)	4 (15.4)	0 (0.0)
Alcohol consumption, *n* (%)	114 (67.5)	65 (67.7)	31 (73.8)	16 (61.5)	2 (40.0)

Abbreviations: *n* = number; ^a^ Mean  ±  standard deviation.

**Table 2:** Questionnaires result in the whole sample and stratified by Tier.

Questionnaires					
	Total (*n* = 169)	Tier 1 (*n* = 96)	Tier 2 (*n* = 42)	Tier 3 (*n* = 26)	Tier 4 (*n* = 5)
*Adherence to the Mediterranean Diet*					
MEDI-LITE	10.6 ± 2.2	10.7 ± 2.2	10.8 ± 2.3	9.9 ± 2.0	10.8 ± 2.2
*Nutrition knowledge*					
GeSNK	72.8 ± 14.9	72.9 ± 14.2	70.2 ± 19.1	78.1 ± 8.4	65.2 ± 11.6
General Nutrition	49.4 ± 7.8	49.6 ± 8.1	48.7 ± 7.2	51.3 ± 6.9	41.8 ± 6.3
Sport Nutrition	24.7 ± 4.8	24.1 ± 5.2	25.2 ± 4.2	26.8 ± 3.0	23.4 ± 5.5
*Eating behaviour*					
HeOr	14.4 ± 5.0	14.2 ± 4.4	13.9 ± 5.8	16.4 ± 5.9	13.4 ± 2.9
OrNe	4.8 ± 3.6	5.0 ± 3.7	4.6 ± 3.5	4.8 ± 3.4	3.8 ± 3.1
EAT-26	4.4 ± 5.2	4.3 ± 5.3	4.4 ± 5.4	5.1 ± 4.9	2.6 ± 1.7
*Sleep quality*					
PSQI	3.8 ± 2.4	3.7 ± 2.3	4.0 ± 3.2	4.1 ± 1.7	3.2 ± 1.1

Data expressed as mean  ±  standard deviation; Abbreviations: *n* = number; GeSNK = General and Sport Nutrition Knowledge Questionnaire; HeOr = healthy orthorexia; OrNe = orthorexia nervosa; EAT-26 = Eating Attitudes Test-26; PSQI = Pittsburgh Sleep Quality Index.

**Table 3:** Dietary habits of the sample population and stratified according to Tier.

Dietary Habits					
	Total (*n* = 169)	Tier 1 (*n* = 96)	Tier 2 (*n* = 42)	Tier 3 (*n* = 26)	Tier 4 (*n* = 5)
*Meal pattern*					
Number of meals per day ^a^	4.3 ± 0.9	4.3 ± 0.9	4.3 ± 1.1	4.4 ± 0.9	4.2 ± 0.4
Regular breakfast consumption, *n* (%)	160 (94.7)	90 (93.8)	40 (95.2)	25 (96.2)	5 (100.0)
Skipping meals, *n* (%)	28 (16.6)	17 (17.7)	6 (14.3)	5 (19.2)	0 (0.0)
Irregular meal timing, *n* (%)	58 (34.3)	32 (33.3)	15 (35.7)	10 (38.5)	1 (20.0)
*Eating speed, n (%)*					
Slow	27 (16.0)	12 (12.5)	11 (26.2)	3 (11.5)	1 (20.0)
Moderate	62 (36.7)	42 (43.8)	12 (28.6)	5 (19.2)	3 (60.0)
Fast	80 (47.3)	42 (43.8)	19 (45.2)	18 (69.2)	1 (20.0)
*Eating out frequency, n (%)*					
≤1 time/week	100 (59.9)	59 (61.5)	23 (56.1)	13 (52.0)	5 (100.0)
2 times/week	44 (26.3)	25 (26.0)	10 (24.4)	9 (36.0)	0 (0.0)
≥3 times/week	23 (13.8)	12 (12.5)	8 (19.5)	3 (12.0)	0 (0.0)
Currently following a nutrition plan, *n* (%)	31 (18.3)	17 (17.7)	9 (21.4)	2 (7.7)	3 (60.0)
Supplement use, *n* (%)	94 (55.6)	47 (49.0)	23 (54.8)	19 (73.1)	5 (100.0)

^a^ Mean  ±  standard deviation; Abbreviations: *n* = number.

**Table 4:** Daily energy and nutrient intake assessed by the 7-day food diary (*n* = 145).

Dietary Intake (7-Day Food Diary)					
	Total (*n* = 145)	Tier 1 (*n* = 86)	Tier 2 (*n* = 35)	Tier 3 (*n* = 22)	Tier 4 (*n* = 2)
Total energy intake (kcal/day) ^a^	2131.7 ± 508.2	2006.3 ± 408.7	2290.6 ± 527.6	2366.2 ± 668.5	2098.7 ± 785.8
Energy availability (kcal/kg FFM/day) ^a^	31.3 ± 8.6	32.8 ± 8.0	31.2 ± 6.5	26.1 ± 11.0	23.4 ± 17.6
Low EA, *n* (%)	25 (17.2)	12 (14.0)	6 (17.1)	6 (27.3)	1 (50.0)
Sub-optimal EA, *n* (%)	75 (51.7)	52 (60.5)	14 (40.0)	8 (36.4)	1 (50.0)
Adequate EA, *n* (%)	45 (31.0)	22 (25.6)	15 (42.9)	8 (36.4)	0 (0.0)
Proteins (g/day) ^a^	96.3 ± 27.9	90.3 ± 24.7	100.3 ± 31.1	111.2 ± 29.3	116.7 ± 18.8
Proteins (g/kg) ^a^	1.4 ± 0.4	1.3 ± 0.3	1.4 ± 0.4	1.5 ± 0.5	1.9 ± 0.3
Proteins (%) ^a^	18.1 ± 3.9	18.0 ± 3.3	17.5 ± 4.4	19.2 ± 4.2	24.3 ± 12.3
Carbohydrates (g/day)^a^	284.2 ± 79.4	268.8 ± 61.4	301.3 ± 84.7	316.5 ± 111.0	281.7 ± 172.1
Carbohydrates (%) ^a^	50.2 ± 5.2	50.4 ± 4.3	50.0 ± 6.5	49.6 ± 5.8	48.0 ± 12.7
Carbohydrates (g/kg) ^a^	4.1 ± 1.2	3.9 ± 1.0	4.3 ± 1.1	4.2 ± 1.5	4.6 ± 2.8
Low CHO availability, *n* (%)	88 (60.7)	42 (48.8)	25 (71.4)	20 (90.9)	1 (50.0)
Sugar (g/day) ^a^	82.3 ± 26.6	78.8 ± 22.8	87.0 ± 25.9	90.0 ± 35.5	58.9 ± 62.8
Sugar (%) ^a^	14.6 ± 3.7	14.9 ± 3.8	14.4 ± 3.5	14.0 ± 2.9	9.1 ± 7.8
Fats (g/day) ^a^	72.5 ± 19.7	68.5 ± 15.7	78.8 ± 24.3	78.6 ± 22.8	69.3 ± 16.7
Fats (%) ^a^	30.7 ± 4.4	30.7 ± 4.1	31.2 ± 4.8	30.1 ± 5.2	30.5 ± 4.2
SFA (%) ^a^	9.8 ± 2.3	9.7 ± 2.2	10.1 ± 2.5	9.4 ± 2.1	11.7 ± 2.6
PUFA (%) ^a^	4.1 ± 0.9	4.0 ± 0.8	4.4 ± 1.3	4.0 ± 0.7	4.5 ± 0.4
MUFA (%) ^a^	14.7 ± 11.5	13.9 ± 2.0	13.6 ± 2.7	19.7 ± 28.9	13.1 ± 1.9
Fiber (g/day) ^a^	27.7 ± 7.6	27.8 ± 7.5	27.3 ± 8.0	28.3 ± 7.1	21.7 ± 14.6
Cholesterol (mg/day) ^a^	223.2 ± 77.5	219.2 ± 67.8	230.7 ± 106.7	226.5 ± 61.7	221.5 ± 48.8
Vit. A (µg) ^a^	737.1 ± 428.1	712.5 ± 411.3	806.1 ± 504.7	733.1 ± 379.6	620.5 ± 266.5
Vit. B9 (folic acid) (µg) ^a^	388.0 ± 109.4	390.7 ± 103.9	381.0 ± 126.1	388.6 ± 108.3	391.6 ± 122.6
Vit. B12 (cyanocobalamin) (µg) ^a^	3.4 ± 1.3	3.4 ± 1.1	3.5 ± 1.7	3.6 ± 1.3	4.4 ± 0.2
Vit. C (ascorbic acid) (mg) ^a^	227.0 ± 167.1	213.2 ± 164.1	259.8 ± 170.4	227.1 ± 179.7	257.1 ± 120.3
Calcium (mg) ^a^	734.7 ± 201.5	712.1 ± 178.4	765.5 ± 236.1	775.4 ± 217.4	708.5 ± 362.7
Phosphorus (mg) ^a^	1480.6 ± 393.0	1451.8 ± 374.7	1396.8 ± 379.9	1714.5 ± 420.8	1594.7 ± 352.6
Potassium (mg) ^a^	3204.5 ± 659.8	3119.4 ± 626.5	3241.7 ± 742.8	3484.7 ± 618.2	3087.1 ± 298.5
Sodium (mg) ^a^	1862.6 ± 614.3	1801.7 ± 546.0	1863.5 ± 679.0	2132.7 ± 711.6	1461.5 ± 606.0
Iron (mg) ^a^	12.8 ± 3.6	12.4 ± 3.0	12.3 ± 3.4	14.8 ± 5.3	14.4 ± 1.6
Zinc (mg) ^a^	10.6 ± 3.4	10.1 ± 3.1	10.7 ± 3.7	12.0 ± 3.7	13.2 ± 6.3
Copper (mg) ^a^	0.9 ± 0.4	0.8 ± 0.4	0.9 ± 0.5	1.0 ± 0.4	0.7 ± 0.6
Water from beverage (L) ^a^	2.2 ± 0.7	2.1 ± 0.7	2.3 ± 0.7	2.2 ± 0.6	3.8 ± 0.8
*Dietary pattern, n (%)* *					
Omnivorous diet	134 (95.0%)	77 (92.8%)	35 (100.0%)	20 (95.2%)	2 (100.0%)
Vegetarian diet	5 (3.5%)	4 (4.8%)	0 (0.0%)	1 (4.8%)	0 (0.0%)
Vegan diet	2 (1.4%)	2 (2.4%)	0 (0.0%)	0 (0.0%)	0 (0.0%)

^a^ Mean  ±  standard deviation; * Dietary pattern data were available for 141 participants (Tier 1, *n* = 83; Tier 2, *n* = 35; Tier 3, *n* = 21; Tier 4, *n* = 2).; Abbreviations: *n* = number; kcal = kilocalories; kg = kilograms; g = grams; EA = Energy Availability; FFM = Fat Free Mass; SFA = Saturated Fatty Acids; PUFA = Polyunsaturated Fatty Acids; MUFA = Monounsaturated Fatty Acids; L = litres.

**Table 5:** Physical activity profile, overall and stratified by Tier.

Physical Activity					
	Total (*n* = 169)	Tier 1 (*n* = 96)	Tier 2 (*n* = 42)	Tier 3 (*n* = 26)	Tier 4 (*n* = 5)
*Mitchell classification, n (%)*					
Low intensity	21 (12.4)	20 (20.8)	0 (0.0)	1 (3.8)	0 (0.0)
Moderate intensity	77 (45.6)	50 (52.1)	20 (47.6)	6 (23.1)	1 (20.0)
High intensity	71 (42.0)	26 (27.1)	22 (52.4)	19 (73.1)	4 (80.0)
*Number of sport practiced, n (%)*					
1	53 (31.4)	40 (41.7)	9 (21.4)	4 (15.4)	0 (0.0)
2	75 (44.4)	47 (49.0)	19 (45.2)	7 (26.9)	2 (40.0)
≥3	41 (24.2)	9 (9.3)	14 (33.4)	15 (57.7)	3 (60.0)
*Primary sport type, n* (%)					
Strength	64 (37.9)	61 (63.5)	2 (4.8)	1 (3.8)	0 (0.0)
Endurance	49 (29.0)	17 (17.7)	16 (38.1)	15 (57.7)	1 (20.0)
Team	20 (11.8)	3 (3.1)	15 (35.7)	2 (7.7)	0 (0.0)
Power	6 (3.6)	1 (1.0)	1 (2.4)	1 (3.8)	3 (60.0)
Racquet	5 (3.0)	4 (4.2)	1 (2.4)	0 (0.0)	0 (0.0)
Combat	4 (2.4)	2 (2.1)	2 (4.8)	0 (0.0)	0 (0.0)
Precision	10 (5.9)	0 (0.0)	5 (11.9)	4 (15.4)	1 (20.0)
Other	11 (6.5)	8 (8.3)	0 (0.0)	3 (11.5)	0 (0.0)
*Training volume ^a^*					
Weekly training volume (h/week)	7.1 ± 3.7	5.3 ± 2.4	8.5 ± 3.1	9.6 ± 3.8	15.4 ± 6.5
Daily training volume (h/day)	1.0 ± 0.5	0.8 ± 0.3	1.2 ± 0.4	1.4 ± 0.5	2.2 ± 0.9
Training frequency (times/week)	5.2 ± 2.3	4.4 ± 1.9	5.9 ± 2.6	6.2 ± 1.8	9.5 ± 0.6
IPAQ (MET-min/week)	8253.4 ± 5470.0	6767.2 ± 3047.2	9677.0 ± 7878.6	10,793.0 ± 6507.4	12,876.2 ± 5102.6
*Injury history, n (%)*					
Previous sport-related injuries	68 (40.5)	30 (31.6)	18 (42.9)	15 (57.7)	5 (100.0)

^a^ Mean  ±  standard deviation; Abbreviations: *n* = number; h = hours; IPAQ = International Physical Activity Questionnaires; min = minutes.

**Table 6:** Anthropometric and body composition characteristics of the sample.

Physical Activity					
	Total (*n* = 169)	Tier 1 (*n* = 96)	Tier 2 (*n* = 42)	Tier 3 (*n* = 26)	Tier 4 (*n* = 5)
Body mass (kg) ^a^	70.7 ± 12.4	69.7 ± 12.5	70.9 ± 13.9	74.5 ± 9.8	67.4 ± 6.6
Height (m) ^a^	1.7 ± 0.1	1.7 ± 0.1	1.7 ± 0.1	1.8 ± 0.1	1.7 ± 0.0
BMI (kg/m^2^) ^a^	23.7 ± 2.9	23.8 ± 3.2	23.3 ± 2.9	24.1 ± 2.1	22.8 ± 1.7
*BMI distribution, n (%)*					
Underweight	4 (2.4)	2 (2.1)	2 (4.8)	0 (0.0)	0 (0.0)
Normal weight	114 (67.5)	63 (65.6)	29 (69.0)	18 (69.2)	4 (80.0)
Overweight	47 (27.8)	27 (28.1)	11 (26.2)	8 (30.8)	1 (20.0)
Obesity	4 (2.4)	4 (4.1)	0 (0.0)	0 (0.0)	0 (0.0)
*Body composition*					
FM% (skinfold) ^a^	18.6 ± 6.6	19.6 ± 6.7	18.1 ± 6.6	16.2 ± 5.8	15.9 ± 5.4
FM% (BIA) ^a^	22.4 ± 7.3	23.8 ± 7.5	20.8 ± 6.8	20.5 ± 6.7	20.7 ± 5.7
Mean FM% ^a^	20.5 ± 6.6	21.8 ± 6.8	19.4 ± 6.2	18.3 ± 6.0	18.3 ± 5.2
FM (kg) ^a^	14.6 ± 5.8	15.3 ± 6.1	13.8 ± 5.9	13.5 ± 4.5	12.2 ± 3.1
FFM (kg) ^a^	56.1 ± 10.8	54.5 ± 10.6	57.0 ± 11.6	61.0 ± 9.9	55.2 ± 7.9
FFM % ^a^	79.5 ± 6.6	78.2 ± 6.8	80.6 ± 6.2	81.7 ± 6.0	81.7 ± 5.2
Rz ^a^	530.5 ± 69.0	542.7 ± 71.1	524.0 ± 62.6	496.6 ± 61.4	528.2 ± 69.1
Xc ^a^	59.9 ± 7.1	60.5 ± 7.7	60.0 ± 6.6	57.0 ± 4.7	62.0 ± 6.6
PhA° ^a^	6.4 ± 0.7	6.3 ± 0.8	6.5 ± 0.6	6.5 ± 0.7	6.6 ± 0.6
*Central adiposity indices*					
Waist circumference adobe the cut-off, *n* (%)	34 (20.1)	23 (24.0)	7 (16.7)	4 (15.4)	0 (0.0)
WHR above the cut-off, *n* (%)	30 (17.8)	19 (19.8)	8 (19.0)	2 (7.7)	1 (20.0)
WHtR above the cut-off, *n* (%)	44 (26.0)	27 (28.1)	11 (26.2)	6 (23.1)	0 (0.0)
Resting energy expenditure (kcal/day) ^a^	1630.1 ± 316.8	1579.8 ± 308.1	1674.6 ± 343.3	1718.4 ± 275.7	1772.2 ± 354.0

^a^ Mean  ±  standard deviation; Abbreviations: *n* = number; kcal = kilocalories; kg = kilograms; m = meters; BMI = Body Mass Index; FM = Fat Mass; FFM = Fat Free Mass; Rz = Resistance; Xc = Reactance; WHR = Waist-to-Hip; WHtR = Waist-to-Height Ratio.

**Table 7:** Relationship between MEDI-LITE score and nutritional, anthropometric, and behavioral variables.

Variable	β Coefficient	95% CI		*p* Value
*Nutrition knowledge*				
GeSNK score	0.04	0.02	0.07	**0.002**
General Nutrition	0.08	0.04	0.12	**<0.001**
Sport Nutrition	0.09	0.02	0.16	**0.009**
*Eating behavior and lifestyle*				
HeOr	−0.02	−0.09	0.05	0.527
OrNe	0.02	−0.07	0.12	0.632
EAT-26	−0.01	−0.07	0.06	0.847
IPAQ	−0.00	−0.00	0.00	0.697
PSQI	−0.03	−0.18	0.11	0.645
*Anthropometry and body composition*				
BMI (kg/m^2^)	−0.02	−0.14	0.09	0.702
Mean FM (%)	0.07	0.02	0.12	**0.011**
FFM (kg)	−0.06	−0.09	−0.03	**<0.001**
WHtR below-threshold vs above-threshold	−0.20	−0.98	0.57	0.608
*Eating habits*				
Breakfast consumption,				
*Yes vs. No*	−0.92	−2.49	0.65	0.251
Skipping meals,				
*Yes vs. No*	0.21	−0.65	1.06	0.637
Consumption of irregular meals,				
*Yes vs. No*	0.18	−0.56	0.91	0.631
Previous dieting,				
*Yes vs. No*	−0.24	−0.93	0.45	0.491
Currently following a nutrition plan,				
*Yes vs. No*	−0.92	−1.86	0.02	0.056
*Energy availability* *	0.04	0.00	0.08	**0.045**
Adequate vs. LEA	0.05	−0.78	0.88	0.904
Sub-optimal vs. LEA	−1.80	−3.49	−0.10	**0.038**

β coefficients were obtained from separate univariable linear regression models. Models were unadjusted for potential confounders or covariates. Bold values indicate statistically significant associations (*p* < 0.05); Abbreviations: GeSNK = General and Sport Nutrition Knowledge Questionnaire; HeOr = Healthy Orthorexia; OrNe = Orthorexia Nervosa; EAT-26 = Eating Attitude Test-26; PSQI = Pittsburgh Sleep Quality Index; BMI = Body Mass Index; kg = kilograms; m = meters; FM = Fat Mass; FFM = Fat Free Mass; WHtR = Waist-to-Height Ratio; LEA = Low Energy Availability; * Energy Availability status: LEA was used as the reference category.

## Data Availability

The data presented in this study are available from the corresponding author upon reasonable request. Although the dataset has been anonymized, it contains detailed individual-level clinical, anthropometric, dietary and lifestyle information collected from human participants. Therefore, requests for data access will be evaluated in accordance with institutional policies, ethical requirements and applicable data protection regulations.

## Abbreviations

The following abbreviations are used in this manuscript:

NK	Nutrition Knowledge
LEA	Low Energy Availability
REDs	Relative Energy Deficiency in Sport
MedDiet	Mediterranean Diet
GeSNK	General and Sport Nutrition Knowledge Questionnaire
TOS	Teruel Orthorexia Scale
HeOr	Healthy Orthorexia
OrNe	Orthorexia Nervosa
EAT-26	Eating Attitude Test-26
EA	Energy Availability
EI	Energy Intake
EEE	Exercise Energy Expenditure
FFM	Fat Free Mass
CA	Carbohydrates Availability
LCA	Low Carbohydrates Availability
PSQI	Pittsburgh Sleep Quality Index
IPAQ	International Physical Activity Questionnaires
BIVA	bioelectrical impedance vector analysis
BMI	Body Mass Index
WHR	Waist-to-Hip Ratio
WHtR	Waist-to-Height Ratio
BD	Body Density
FM	Fat Mass
R	Resistance
Xc	Reactance
PhA°	Phase Angle
REE	Resting Energy Expenditure
VO_2_	oxygen consumption
VCO_2_	carbon dioxide production
RQ	Respiratory Quotient
